# Platelets and Their Role in Immunity: Formation, Activation and Activity, and Biologically Active Substances in Their Granules and Extracellular Vesicles

**DOI:** 10.1155/ijin/8878764

**Published:** 2025-09-22

**Authors:** Beata Tokarz-Deptuła, Łukasz Baraniecki, Joanna Palma, Michał Stosik, Anhelli Syrenicz, Roman Kołacz, Wiesław Deptuła

**Affiliations:** ^1^Institute of Biology, Faculty of Physical, Mathematical and Natural Sciences, University of Szczecin, Szczecin, Poland; ^2^Doctoral School, University of Szczecin, Szczecin, Poland; ^3^Department of Biochemical Sciences, Pomeranian Medical University in Szczecin, Szczecin, Poland; ^4^Institute of Biological Science, Faculty of Biological Sciences, University of Zielona Góra, Zielona Góra, Poland; ^5^Department of Endocrinology, Metabolic Diseases and Internal Diseases, Pomeranian Medical University in Szczecin, Szczecin, Poland; ^6^Institute of Veterinary Medicine, Faculty of Biological and Veterinary Sciences, Nicolaus Copernicus University in Toruń, Toruń, Poland

## Abstract

When presenting the role of platelets in immunity, the organelles and processes occurring within them are listed, and due to their lack of a cell nucleus, their specific transcriptional activity is discussed. Their formation, activation, and functional activity are also described, along with the characterization of elements important for shaping their intravascular activity, including immunity, such as their granules and extracellular vesicles (EVs). Presenting platelets in the context of their immune role, it is indicated that their activation and activity are complex processes resulting from the binding of their receptors with the endothelium of blood vessels, pattern recognition receptors (PRRs) of immune system cells, pathogen-associated molecular patterns (PAMPs), damage-associated molecular patterns (DAMPs), and lifestyle-associated molecular patterns (LAMPs). As a result of these interactions, the inflammatory phenotype of platelets is promoted, making them not only the fundamental elements of homeostasis in blood vessels but also, above all, of immunity. Discussing the immunological role of platelets in blood vessels, biologically active substances contained in their five types of granules (α, δ, lysosomes, peroxisomes, and T), and two subtypes of EVs (exosomes and ectosomes—microvesicles) that determine their activity, including the immunological status, are characterized. Moreover, describing the role of platelets in blood vessels, it has been demonstrated that these cells are not only effective sentinels and regulators of these vessels, as previously assumed, but also exhibit strong pro- and anti-inflammatory, immunomodulatory, and regenerative effects, making them fundamental cellular elements determining intravascular immunity. It is also pointed out that by inducing an inflammatory environment in blood vessels, platelets can not only cause potential tissue damage but also emerge as potential cellular candidates for treating inflammatory diseases.

## 1. Introduction

**Figure 1:**
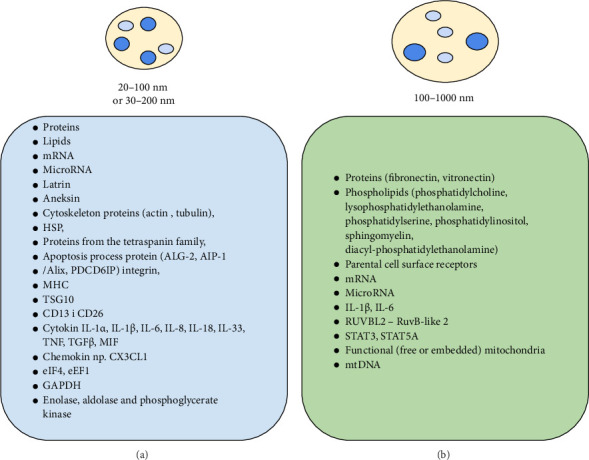
Selected biologically active substances found in two subtypes of platelet EVs, i.e., (a) exosomes and (b) ectosomes—microvesicles [1, 2, 10, 16, 20–42]. Abbreviations: HSP, heat shock proteins; PDCD6IP (ALG-2 [apoptosis-linked gene-2], AIP-1/Alix-ALG-2-interacting protein X), programmed cell death 6-interacting protein; MHC, major histocompatibility complex; TSG10, tumor susceptibility gene 10; CD, cluster of differentiation; IL, interleukin; TNF, tumor necrosis factor; TGFβ, transforming growth factor beta; MIF, macrophage migration inhibitory factor; CX3CL1 (C-X3-C), motif chemokine ligand 1; eIF4, eukaryotic initiation factor 4F; eEF1, eukaryotic initiation factor 1F; GAPDH, glyceraldehyde-3-phosphate dehydrogenase; mRNA, messenger RNA; RUVBL2, RuvB-like 2–RuvB-like AAA ATPase 2; STAT, signal transducer and activator of transcription; mtDNA, mitochondrial DNA.

Platelets in mammals, including humans, are morphological components of the blood characterized by a discoid shape with a diameter of 2–5 μm [1–3], whose role was previously associated with intravascular homeostasis but is now also linked to immunity [4–7]. These cells exhibit typical elements and processes found in mammalian cells but lack a nucleus and do not undergo de novo transcription [1, 3]. In their cytoplasm are ribosomes for protein translation and 5–8 mitochondria for energy production, which also contain genetic material “enriched” with transcription factors (TFs) that interact with the regulatory sequences of their genes [8, 9]. Despite lacking a nucleus, their transcriptome consists of approximately 9500 RNA transcripts (mRNA), various classes of noncoding RNA, antisense transcripts to protein-coding loci, and tRNA and microRNA [10]. This composition of the platelet transcriptome determines its essential transcriptional functions, as it can “translate” it into proteins, including specific substances involved in intravascular homeostasis and immunity, both under physiological and pathological conditions [7, 11]. It is suggested that, in the case of platelets, there is uncertainty regarding whether translation regulation on the platelet mRNA template occurs via microRNA, as it has been shown that a large amount of microRNA influences this process and may even inhibit it [10]. It has been found that the platelet microRNA repertoire consists of over 500 sequences, and the modification at the 3′ end results in adenylation, ensuring microRNA stability, or uridylation, leading to the degradation of this molecule [12, 13]. Thus, although mammalian platelets, including human platelets, contain the essential elements for the biogenesis of these cells' functions, such as cytoplasmic protein components of the RNA pathway, including Dicer enzymes, thrombocytopenia-absent radius RNA-binding protein (TAR), argonaute 2 endonuclease (Ago2), and other components, the absolute role of microRNA is unequivocally emphasized [10]. It should also be added that the platelet transcriptome is “enriched” with over 1600 TFs, which, by binding to the promoter or regulatory sequences of genes, control the specific transcription of genetic information from DNA to messenger RNA in these cells [14]. It is also associated with the fact that the TFs responsible for the transcription process act independently of the classical transcriptome programs and are represented by, among others, RUNX Family Transcription Factor 1 (RUNX1), GATA Binding Protein 1 (GATA1), nuclear factor Kappa B (NFκB), peroxisome proliferator-activated receptor gamma (PPARγ), and nuclear factor of activated T-cells (NFAT) [9, 14, 15]. Another distinctive feature of platelets, besides the lack of a nucleus and de novo transcription [1, 2, 16], is that their skeleton exhibits a unique integrity that remains unchanged even under the influence of forces generated by blood flow in vessels [17–19]. Furthermore, among these platelet characteristics is the presence of three types of microtubules in their cytoplasm, as well as a very rich set of their extracellular and intracellular receptors, which participate in the activation and activity of these cells [7, 11]. In this panel of very specific platelet features, there is also the presence of five types of granules in their cytoplasm (Table 1) and two subtypes of extracellular vehicles (EVs) (Figure 1), equipped with an abundant set of biologically active substances that condition, influence, and determine the maintenance of intravascular homeostasis and immunity. These biologically active substances found in these organelles, which define the role, including the immune role of platelets in blood vessels, have led to the distinction of two subpopulations, namely, platelets that aggregate, causing mechanical retraction of the clot, and platelets that exhibit procoagulant activity [20, 43]. It should also be added that among the rich array of biologically active substances found in platelet granules (Table 1), the presence of serine proteases–granzymes (with no indication of which granules) has been registered, showing both intracellular and extracellular activity, including interactions with the extracellular matrix [44].

## 2. Platelet Formation

Platelets are produced in the bone marrow from cells of the myeloid series through the process of thrombopoiesis as the final element of differentiation and fragmentation of megakaryocytes, representing the mature form of the platelet-producing or megakaryocytopoietic system [1, 45]. Megakaryocytes, from which platelets derive, are cells ranging in size from 50 to 100 μm, possessing a characteristic multilobed nucleus, which constitutes “distinct” nuclei [46], and arise from progenitor megakaryocytic cells in a three-step process regulated by thrombopoietin (TPO), IL-1α, IL-3, IL-6, IL-11, and microRNA, as well as other molecular regulators [47, 48]. In the first maturation stage, megakaryocytes are small, immature cells with highly basophilic cytoplasm and a high nucleus-to-cytoplasm ratio. In the second maturation stage, they change the nucleus-to-cytoplasm ratio as the cytoplasm becomes more expansive and less basophilic, containing single azurophilic granules. In the third stage of maturation, megakaryocytes have a spacious, lightly pink cytoplasm arranged centrally, with an extensive system of three types of tubules and numerous granules. From this third stage of megakaryocyte development, through the elongation and reorganization of the cytoplasm and the presence of demarcation membranes, proplatelets (megakaryocyte protoplatelets) are produced, which, upon entering the bloodstream, become platelets [1, 8, 49]. It has been shown that from a single megakaryocyte, due to the polyploidy of its genetic material, 2000–10,000 platelets can be produced, as megakaryocytes replicate their DNA without cell division during the process of endomitosis [1, 50, 51]. In specific cases, the release of platelets from megakaryocytes may occur due to their rupture [7, 8, 52]. During thrombopoiesis, platelets are “loaded” with, among others, major histocompatibility complex (MHC) class I antigens, through which they interact with hematopoietic stem cells (HSCs), toll-like receptors (TLRs), through which they interact with pathogen-associated molecular patterns (PAMPs), and markers from the purinergic subfamily P2Y_1_ and P2Y_12_ [24, 53–55]. These last receptors, by binding to, among others, mitochondria and the inflammasome of platelets, transfer immunological information to “proplatelets” and platelets. It is also indicated that platelets can be generated by extramedullary megakaryocytes, including those in the lungs [56, 57]—distinct from bone marrow derived [22, 56], although it has been registered that megakaryocytes are also found in lymph nodes, spleen, and liver [22, 58]. They exhibit distinct morphological, functional, and transcriptomic properties depending on their origin. [8, 58]. It has been shown that megakaryocytes derived from the lungs possess atypical MHC class II molecules involved in immune surveillance, while megakaryocytes derived from the spleen have high expression of the CD40 ligand (CD40L), through which they support the formation of NETs [22].

The number of platelets in the bodies of mammals, including humans, changes with age and in humans, ranges from 125,000 to 400,000 cells/μL, making them the most numerous group of blood cells after erythrocytes [15, 45]. It is assumed that approximately 100 billion platelets are produced daily in humans, which results in their number being estimated at 1–6 × 10^13^ cells, with a surface area of 1–7 m^2^ [22, 59, 60]. This results in a considerable surface area, along with the cells of the subendothelial layer of blood vessels and immune cells in the blood, which requires a huge strategy for their surveillance [7, 61]. In human blood, platelets account for 60%–70% of the entire platelet pool, 30% in the spleen, and only 5% in the bone marrow and lungs, with their “lifespan” estimated at 5–10–12 days [1, 7]. In physiological conditions in mammals, including humans, the primary activators of thrombopoiesis are TPO, IL-1α, IL-3, IL-6, and IL-11, as well as microRNA. In pathological conditions, such as bacterial infections, lipopolysaccharide (LPS) acts as an activator [7, 56, 62]. In the case of viral infections, TPO is an activator of this process, but it can also impair it and even lead to its inhibition [6, 46, 62]. It has been shown that during both bacterial and viral infections, inflammatory cytokines such as TNF-α, IL-1β, IL-3, IL-6, IL-8, and IL-11 also influence the process of thrombopoiesis [6, 46, 62]. The process of thrombopoiesis in mammals, including humans, is also influenced by the gastrointestinal tract's microbiome (bacteria, viruses, and fungi). It has been shown that dysbiosis in this ecological niche, through its effect on T lymphocytes, mainly Th and Tfh, can lead to immune thrombocytopenia (ITP) [63, 64]. Moreover, thrombopoiesis in humans is also influenced by the aging process of the microorganism, as it is associated with decreased activity of HSC and TPO, as well as a reduced content of mRNA and ribosomal RNA [45, 65].

## 3. Activation and Activity of Platelets

The activation of platelets occurs as a result of interactions with various endogenous and exogenous factors, and it is a complex process that involves the engagement of their broad repertoire of extracellular and intracellular receptors [3]. It leads to their activation and the initiation of activity in functions such as movement, aggregation, and adhesion, as well as degranulation and the secretion of biologically active substances from their granules and EVs [2, 3, 6, 21]. It has also been shown that factors triggering activation and influencing the activity of these cells, as well as vascular cells and immune cells, create a specific role for platelets, making them potential cellular elements also used in the therapy of inflammatory background diseases [6, 66]. As a result of platelet activation, through the binding of their receptors with markers of pattern recognition receptors (PRRs) on immune cells, such as receptors activating the phagocytosis process, TLRs, and NOD-like receptors (NLRs), as well as PAMPs, damage-associated molecular patterns (DAMPs), and lifestyle-associated molecular patterns (LAMPs), the inflammatory phenotype of these cells is promoted [53, 67]. It is accepted that the process of platelet activation at the molecular level is mainly associated with the cyclic GMP–AMP synthase (cGAS)-stimulator of interferon genes (cGAS-STING) pathway, which is “transferred” from megakaryocytes. This pathway activates TFs associated with, among others, the stimulators of type I interferon gene synthesis [46]. This condition leads to the formation of a Soluble N-ethylmaleimide sensitive factor Attachment Protein REceptor (SNARE) protein complex, which results in the degranulation of platelet granules [6]. This pathway of platelet activation should also include their stimulation through signals dependent on Src kinase (proto-oncogene tyrosine-protein kinase Src), which affects the phosphorylation of the inhibitory motif of the immunoreceptor ITSM, enhancing their interaction with leukocytes [6]. Furthermore, platelet activation is also associated with neuronal guidance proteins (NGPs), such as Sema 3A, 7A, 4D, EphA4, B1, and Slit 2, which additionally create signals that stimulate and inhibit the recognition of axons by neurons, thus involving peripheral neurons of the macroorganism in this process [49]. Regardless of the mechanism of platelet activation, each time this process occurs, there is an intracellular flow of calcium and the translocation of negatively charged phospholipids, which aids, with the help of tissue factors, the activation of coagulation factors and may also contribute to the formation of thrombosis [22, 24, 49]. It has been described that in cases of platelet interactions with DAMPs commonly arising from injuries in mammals, including humans, an increase in their activity is recorded, primarily in terms of adhesion and aggregation—processes important in the pathogenesis of thrombosis [5, 11, 46]. It has been shown that after stimulation of platelets by DAMPs, through their integrin receptors, large amounts of reactive oxygen species (ROS) are released, which very effectively enhance intravascular immune responses against infections [6, 11, 46]. Moreover, as a result of the interaction between platelets and Kupffer cells in the liver, as well as gut cells that convert serotonin to 5-hydroxyindoleacetic acid (5-HIAA), there is an intense activation of the signaling pathway of platelets, which has been recorded in patients affected by systemic lupus erythematosus (SLE) [6]. In the case of interactions through purinergic receptors P2Y1 and P2Y12 of platelets with endothelial cells of damaged blood vessels, which are characterized by exposed collagen and the release of free radicals and soluble agonists (ADP, thromboxane A2 [TxA2], von Willebrand factor [vWF], and fibrinogen), there is an increase in the aggregation activity of these cells [24, 46, 68]. A similar effect has been recorded following the interaction of the platelet receptor intercellular adhesion molecule 1 (ICAM-1) and the integrin marker αIIβ3 with the glycocalyx of endothelial cells [17, 61]. The interaction of platelets through protease-activated receptors (PAR) and integrin markers with thrombin leads to an increase in TxA2 and disintegrin and metalloproteinase domain-containing proteins 10 (ADAM10) and 15 (ADAM15) from the matrix metalloproteinase (MMP) family, increasing their secretory activity [69–71]. Moreover, it has been shown that the interaction of platelets with endothelial cells, as well as with PAMPs, DAMPs, and LAMPs, also influences the rearrangement of their cytoskeleton and receptors, leading to the development of a proinflammatory platelet phenotype and the creation of a proinflammatory environment. [6, 22, 46]. It has also been recorded that the increased activity of platelets, mainly in terms of aggregation, is enhanced by the “transition” of their integrin receptors GPIIb/IIIa from a low-affinity state to a high-affinity state [7], which leads to their aggregation and the formation of a clot. This element also limits the spread of microbiological factors in blood vessels [3, 7, 43]. It has also been described that platelet aggregation increases after binding their undefined receptors with epinephrine, ADP, and the nongenomic TF NFAT [9, 49]. Their interaction with PAMP, DAMPs, and LAMPs activates the phagocytosis of PMN cells and the activity of T and B lymphocytes [22, 46, 53]. It should be added that the process of platelet activity is also associated with a shape change, from a disc-like to a spherical form with numerous pseudopodia, which increases their surface area for interaction with other cells, including immune cells, thereby enhancing their intravascular immune potential [22, 24, 49]. It should be added that the process of platelet activity is also associated with a shape change, from a disc-like to a spherical form with numerous pseudopodia, which increases their surface area for interaction with other cells, including immune cells, thereby enhancing their intravascular immune potential [22, 49, 72]. It has been shown that coronins of class I influence the dynamics of platelet activity, coronins of class II affect the rearrangement of their cytoskeleton, while coronins of class III interact with elements of their cytoplasm, mainly the Golgi apparatus. It should also be noted that the changing shape of platelets is associated with the emergence of numerous pseudopodia (lamellipodia and filopodia), which expands their ability to interact with endothelial cells, immune system cells, as well as PAMPs, DAMPs, and LAMPs [7, 22, 53]. The characterized state of platelets, manifesting their increased activity, is associated with the action of biologically active substances secreted from their granules and EVs, which directly affect the activity of immune cells [22, 23] and through an indirect route, affect the expression of their genes, which leads to increased killing of PMN and MN cells [7, 21, 73], including the formation of NETs [7, 62, 74]. Such an interaction between platelets and PMN and MN cells also affects hematopoietic progenitor cells (HPCs) and antigen presentation by immunocompetent cells, leading to an increased synthesis of various cytokines, including IFN, TNF-α, and IL-6, as well as immunoglobulins [22, 73, 75]. It should be added that the activity of platelets, resulting from their interaction with PMN, MN, as well as dendritic cells (DC), basophils, eosinophils, and T and B lymphocytes, also increases their immunological activity, particularly in terms of their aggregation—a function that is important in thrombosis [6, 11, 22]. It should be noted that the immunological activity of platelets in blood vessels is “dynamically built” by them, as it is associated with the release of biologically active substances from their granules and EV, which interact with the cells of the blood vessel wall and immune system cells residing in the vessels [7, 22, 23, 56]. It should also be added that reduced platelet activation has been described as a result of the binding of their receptors with PAMPs from *Escherichia coli* and *Staphylococcus aureus*. This reaction leads to the degradation of their antiapoptotic proteins, Bcl-xL, and increases their apoptosis, thereby reducing their number and, as a result, lowering the defensive potential of platelets in the blood vessels [58, 59, 76]. A similar status has been demonstrated in the case of a *Dengue* virus infection, which lowers their bactericidal capacity by increasing platelet apoptosis and reducing the activity of their mitochondria [7, 46, 62]. The immunological activity of platelets also includes their role in lymphatic vessel development, as it is linked to the fact that activation of the platelet receptor C-type lectin-like receptor 2 (CLEC-2) by podoplanin leads to the separation of lymphatic vessels from blood vessels during embryonic development [31, 77]. The presented anti-infectious, activating, immunomodulatory, and hematological activity of platelets in blood vessels arises from their interaction with endothelial cells, blood cells of the immune system, as well as PAMPs, DAMPs, and LAMPs, is related to not only their continuous surveillance of the blood vessel walls but also their monitoring of the “inflammatory homeostasis”—that is, sealing microinjuries in the vessels by platelets without forming a clot [6, 7, 62]. This last reaction, physically associated with sealing the damaged endothelial cells of blood vessels by platelets, is linked to the full integrity of blood vessels, resulting from the binding of the glycoprotein receptors GPIb-IX-V on these cells with vWF. It also influences the blood clotting process and clot formation, a state that also contributes to the immunological potential within blood vessels [43, 78, 79]. It has been recorded that to prevent excessive platelet aggregation during the “activation” of platelets, which could lead to states causing intravascular coagulation and vessel occlusion, platelet aggregates “migrate” onto PMN [16, 31]. It is accepted that the characterized platelet activity, related to their continuous surveillance of the vascular endothelium rich in glycocalyx, as well as their constant contact with circulating immune cells in the bloodstream, makes these cells “touch-and-go” “patrolling agents,” shaping good homeostasis and a high state of intravascular immunity [7, 11, 76]. It causes platelets to be described as effective “guardians” of blood vessels and intravascular regulators of homeostasis. They are important cellular elements responsible for pro- and anti-inflammatory actions, immunomodulation, and regeneration within blood vessels, as well as playing a role in mechanisms that prevent damage to organs and tissues [11, 60]. This role of platelets is also associated with their action in preventing inflammation resulting from infection and leading to sepsis or septicemia, making these cells an important element in preventing thrombosis and sterile inflammation [5, 22, 80]. It should also be added that the role of platelets in infections, arising from their dysfunction in processes such as “sweeping” and destroying pathogens (e.g., through phagocytosis and bactericidal activity), is integral to their crucial role in health and disease in mammals, including humans [7, 11].

## 4. Platelet Granules—Important Elements Determining Homeostasis and Intravascular Immunity

Platelets contain five types of granules: α, δ (osmophilic and dense), lysosomes (λ granules), peroxisomes, and T granules (Table 1). These granules differ in the biologically active substances they contain and thus in their functions. The number of granules in platelets is estimated to be over 300 [56, 65], although according to Cognasse et al. [81], there are more than 1000 substances within these granules. These biologically active compounds in platelet granules exert activating, modulatory, regulatory, and stabilizing effects on the vascular environment, including leukocytes and platelets. It has been demonstrated, for instance, in thrombosis, as a key element in detecting and repairing damage, including vascular damage, through substances such as cytokines like TGF-β and PGGF [7, 68]. It has been shown that the process of platelet granule degranulation and the release of biologically active substances occur as a result of the binding of their soluble protein N-ethylmaleimide (NEM) with the SNARE protein complex [82, 83]. This state leads to the fusion of platelet granules with their cell membrane, resulting in granule degranulation and the release of biologically active substances via exocytosis [82]. During the exocytosis process, the membranes of platelet granules may also fuse with the membranes of their open canaliculi, further enhancing the release of the contents from the platelet granules [2, 52]. It has been shown that the process of platelet granule degranulation and exocytosis of biologically active substances from these cells occurs after their activation through receptors, including particularly the protease-activated receptor 4 (PAR4) [3, 7, 84] triggered by factors such as infection, autophagy, as well as interactions with thrombin, ATP, and epinephrine [85, 86]. It has been demonstrated that the time of granule degranulation and exocytosis of the granule contents in platelets depends not only on the type of factors acting on them but also on the type of granules and the nature of the biologically active substances contained within them [52, 87]. It has been shown that the degranulation process occurs most rapidly in δ granules (osmophilic and dense) and α granules, while it occurs most slowly in lysosomes (λ granules) and peroxisomes [86, 88], and the degranulation of T granules is still poorly understood [52].

### 4.1. Biologically Active Substances of Platelet α Granules in the Aspect of Intravascular Immunity

The α granules of platelets range in size from 200 to 500 nm and are the most numerous, with 40–80 per platelet, accounting for as much as 10% of the volume of these cells [89, 90]. These granules contain: growth and mitogenic factors, coagulation proteins, cytokines, chemokines including kinecidins, adhesion molecules, inhibitors, and other compounds (Table 1). These substances affect the homeostatic function of platelets, including a regulatory effect, including activating, stimulating, and stabilizing on the endothelial cells of blood vessels and blood cells, including cells of the immune system, thereby determining not only intravascular homeostasis but also the immune status in the vessels [7]. It has been recorded that among the growth factors present in the α granules of platelets (Table 1), the transforming growth factor β (TGF-β) influences the differentiation of, among others, Treg and Th17 lymphocytes and affects the activity of APC antigen-presenting cell (APC) RAR-related orphan receptor gamma (RORγT), which is an important factor in regulating, among other processes, IgA isotype switching, primarily in the gastrointestinal tract. These proteins shape the homeostasis status and influence the microbiota of this biotope. [62, 91]. Moreover, TGF-β is also involved in epithelial-mesenchymal transition (EMT) in cancer cells [28, 60] and affects the proliferation of mesangial cells in the process of fibrosis in the skin, heart, kidneys, and lungs [22]. These processes can be suppressed through the interaction of Treg lymphocytes with TGF-β and IL-10, leading to the reprogramming of macrophages into an anti-inflammatory phenotype, which is utilized in the therapy of inflammatory background diseases, including autoimmune diseases [6, 22]. On the other hand, the mitogens present in α granules (Table 1) influence angiogenesis, as vascular endothelial growth factor (VEGF) affects vessel development, platelet-derived growth factor (PDGF) and granulocyte-macrophage colony-stimulating factor (GM-CSF) affect platelets and granulocytes, hepatocyte growth factor (HGF) affects liver cells, fibroblast growth factors (FGFs) affect fibroblasts, and insulin-like growth factor (IGF) affects pancreatic cells and the lifespan of mammals. Recent studies [6, 20, 24] have shown that the main compounds influencing angiogenesis among the mitogens are VEGF, PDGF, and TGF-β growth factors. On the other hand, the coagulation proteins of these granules (Table 1) determine intravascular homeostasis. In contrast, cytokines, chemokines including kinecidins (killer chemokines), adhesive molecules, inhibitors, and other molecules of these granules shape the immune status in the blood [7, 62]. Cytokines of these platelet granules shown in Table 1, by influencing the immune status in blood vessels, mainly exert an activating effect on endothelial cells of blood vessels and cellular elements of the immune system. Cytokines in these granules, among others, through matrix metalloproteinase 2 and 9 (MMP-2 and MMP-9), enhance the immune status in blood vessels by influencing the increased formation of platelet-leukocyte aggregates [20, 24]. Moreover, it has been shown that TNF type I cytokine is not only a regulator of the inflammatory response, including in patients with SLE [20, 24], but also influences the formation of proplatelets and platelet count, as it affects liver functions related to the production of TPO [46]. Chemokines (Table 1), including CXCL8 (IL-8) CXCL4 (PF4: platelet factor), also occur in these granules and, due to changes in the expression of their adhesion molecules and the release of lysosomal enzymes by them, they cause increased chemotaxis and killing of neutrophils and the formation of NETs. It has been shown that chemokines such as CXCL4, CCL3 (macrophage inflammatory protein-1-alpha [MIP-1-α]), CXCL5 (epithelial-derived neutrophil-activating protein 78 [ENA78]) and kinecidins CCL5 (RANTES) (Table 1), not only affect the recruitment of leukocytes, including neutrophils to the sites of vascular “infiltration,” but also activate them because by “depositing” on the surface of vascular endothelial cells, they activate leukocytes in terms of their migration. Moreover, the chemokine CXCL5 and the antimicrobial chemokine kinocidin CXCL7 (NAP-2) of these granules (Table 1), together with chemokines and thrombocins derived from carboxyl-terminal deletions, exhibit strong bactericidal activity. However, recently [21], it has been shown that kinecidins do not fully demonstrate protective activity during infection. It has also been recorded that chemokines such as CXC7 (NAP-2), CCL2 (MCP1), and CCR7 (MCP3), by showing chemotactic activity toward plasmacytoid DC cells (pDC) synthesizing IFN-α, activate the development of the inflammatory process, as well as the formation of platelets [46, 92]. It has been shown [93] that the chemokine CXCL4, by reducing the expression of proinflammatory factors such as TNF, complement component C1q and the microglia activation marker in the hippocampus, reduces the circulating levels of the chemokine CCL2, cyclophilin 2, and TNF, which reduces the aging process of the organism, among others by reducing the number of Tc lymphocytes and exhausted lymphocytes [93]. It has also been recorded that the platelet chemokine PF-4 and β-thromboglobulin, as well as the growth factor TGF-β (Table 1), by increasing platelet activity, sensitize them to DAMPs commonly generated during injury [5]. On the other hand, the adhesive molecules present in α granules (Table 1) are compounds involved in, among other things, clot formation and the development of inflammation, as they recruit leukocytes with phagocytic and cytotoxic properties through P-selectins, which act destructively on bacteria and viruses. It should also be noted that thrombospondin, an adhesive molecule of these granules (Table 1), is an alternative adhesion substrate for the vWF, which may inhibit the phagocytic activity of MN cells. The characterized adhesive particles promoting platelet aggregation, among other things, through the interaction of their integrin receptor GPIbα with thrombospondin and TGF-β (Table 1), significantly enhance the immunological role of platelets. It has also been shown that the tissue inhibitors of metalloproteinases (TIMPs) and disintegrins of MMP—ADAM10 (Table 1), present in these α granules, interact with endothelial cells and blood leukocytes, contributing to the establishment of “good” homeostatic conditions in blood vessels. Meanwhile, other molecules present in the α granules of platelets, such as defensins, antimicrobial peripheral myelin protein (PMP), β-lysin, and immunoglobulins (Table 1), elevate the immune status in the blood, primarily in terms of antiviral and antibacterial immunity [7, 60]. Moreover, among these other molecules in the α granules of platelets (Table 1), calprotectin and high mobility group box 1 (HMBG1) are substances that, together with mitochondrial DNA (mDNA) derived from the EVs of these cells, enhance the activity of pDCs and neutrophils and increase their interaction with platelets. It has also been shown that upon oxidation of the HMBG1 component from the α granules of platelets, this compound stimulates the formation of NETs, which may contribute to thrombosis formation [22].

### 4.2. Biologically Active Substances of δ Granules (Osmophilic and Dense) of Platelets in the Aspect of Intravascular Immunity

Platelet δ granules are slightly smaller than α granules, with sizes ranging from 150 to 300 nm; they occur in smaller numbers, with 3–8 of them per platelet, and constitute only 1% of the volume of these cells [7, 94]. It has been shown that the amines and mediators present in these granules, including thrombin, serotonin, histamine, and other molecules such as nucleotides—ATP, ADP, and GDP, as well as Ca and Mg ions, P-selectin, and platelet-derived protein 1 and 2 (Table 1), like substances found in α granules, strongly influence the shaping of homeostasis in blood vessels and immunity. These compounds are referred to as mediators of vascular tone. Studies [7] have shown that the amines and mediators in these granules, such as serotonin, histamine, and catecholamines, primarily affect blood vessels by influencing their volume and constriction and the recruitment of immune cells. It is noted that although serotonin activates T lymphocytes, including naive T cells and CD4+ T cells, it may reduce neutrophil migration. Meanwhile, the nucleotides ATP, ADP, and GDP (Table 1) present in these granules condition platelet movement through the ligation of purinergic receptors (P2X for ATP and P2Y for ADP) and also influence the differentiation of immune cells. Additionally, through the inflammasome pathway, they activate mitochondria and cytokine synthesis and release [6, 46, 54, 55]. Among other compounds contained in δ granules (Table 1), it has been shown that glutamines participate in immune processes. In contrast, the platelet-derived proteins 1 and 2 in these granules exert a destructive effect on pathogens such as *Bacillus subtilis*, *Escherichia coli*, *Staphylococcus aureus*, *Lactococcus lactis*, *Cryptococcus neoformans*, and *Plasmodium falciparum*. It makes the substances in these granules, along with those from other granules, place platelets as crucial cellular components of immunity within blood vessels, both in physiological and pathological conditions—caused by bacterial and viral infections as well as autoimmune diseases [6, 7, 95]. Moreover, it has been shown [20, 96] that the deficiency of certain substances in δ granules of platelets, characterized by oculocutaneous albinism and pneumonia, is the cause of Hermansky–Pudlak syndrome.

### 4.3. Biologically Active Substances of Lysosomes (λ Granules) and Peroxisomes in the Aspect of Intravascular Immunity

The granules of platelets, referred to as lysosomes (λ granules) and peroxisomes, are represented more modestly compared with α and δ granules, with only a few per platelet, and are also much poorer in biologically active substances (Table 1). It has been shown [7] that glycohydrolases in lysosomes and catalase and peroxidase in peroxisomes affect intravascular immunity, but to a lesser extent than the substances found in α and δ granules. It is noted that catalase and peroxidase in the peroxisomes of these platelet granules are substances with bactericidal, including oxidizing, actions and represent an important element of the oxygen-dependent immune cell killing system—a highly effective system in combating bacterial, viral, and even parasitic infections. Furthermore, studies by Almeida et al. [76] have shown that substances contained in these granules, under high thrombin concentrations, strongly influence the activity of endothelial cells of blood vessels and immune cells in the blood, significantly enhancing intravascular immunity and affecting clot dissolution [21].

### 4.4. Biologically Active Substances of Platelet T Granules in the Aspect of Intravascular Immunity

These granules have been recently described, and it has been recorded that they exclusively contain the intracellular receptor TLR-9 and specific membrane proteins unique to these granules [97]. From these studies, it follows that the former proteins' condition the participation of platelets in viral infections and intracellular bacterial infections, while the latter exert an activating effect on cellular immune elements in the blood, namely, leukocytes and platelets.

## 5. EVs of Platelets–Key Elements Conditioning Intravascular Homeostasis and Immunity

EVs of platelets, previously referred to as membrane microvesicles (MVs), are a term that refers to membranous structures that have a lipid bilayer, do not replicate on their own, and are released by various cells of the macroorganism, including bacteria [2, 98, 99]. According to the International Society for Extracellular Vesicles [30, 99], the recommended term for EVs, as well as a nonvesicular extracellular particle (NVEP), is an extracellular particle (EP) [30, 99]. EVs were first recorded in 1946 by Chargaff and West [100] in human plasma as procoagulant particles originating from platelets, and in 1967, Wolf [101] described them in human blood as an element also produced by platelets with procoagglutinating activity and called them “platelet-dusts.” EVs are small, heterogeneous extracellular structures with a diameter ranging from 20 to 1000 nm, enclosed by a bilayer lipid membrane. They do not replicate, lack a nucleus, and do not possess specific markers [98, 99]. Two subtypes have been described within them (Figure 1); exosomes and ectosomes or MVs [102]. The classification of EVs into these two subtypes (Figure 1) is based on their biogenesis [41, 103]; however, in practice, their classification is primarily determined by their biological, including biochemical, properties [40, 102]. It should be added that according to the ISEV guidelines from 2024 [99], regarding the names of these structures, their terminology requires great caution, although Wizner et al. [37] and Qiu et al. report that not only exosomes and ectosomes are distinguished within EVs, but also MVs, apoptotic bodies and migratory bodies are distinguished in the latter. Moreover, Wizner et al. [37] report that EVs, depending on the type of cell they originate from, can range in size from 100 to even 5000 nm, and their names are related to the tissues from which they originate because, for example, apoptotic bodies originating from cancer cells are referred to as oncosomes.

Regardless of the data on the names of EVs (exosomes, ectosomes–MVs), it is assumed that in mammals, including humans, these structures, in addition to blood, occur in saliva, milk, bile, urine, feces, semen, synovial, and cerebrospinal fluid, and secretions from the nose, bronchi, and uterus [23, 33, 98]. It is also indicated that EVs, both in physiological and pathological conditions, originate, among others, from cellular morphological elements of blood [23, 98], including as much as 70%–90% from platelets [37, 104]. It is assumed that the process of EVs formation and release from platelets in physiological conditions occurs, among others, as a result of the action of physiological platelet agonists/mediators (ADP, thrombin, and chemokines) [2, 10, 60], while in pathological conditions, among others, during infection, oxidative stress, inflammation, apoptosis, and neoplastic processes [39, 68, 105, 106]. It has also been shown that EVs enter the target cell via membrane fusion, i.e., endocytosis, phagocytosis, or both [37, 104]. These structures, derived from platelets, are rich in cell membrane proteins and cytoplasmic membrane proteins of these cells, and their components (Figure 1) determine homeostasis and intravascular immunity, including anti-infective immunity and intercellular communication, and are also used in the diagnosis of cancer, autoimmune, allergic, and even mental diseases [11, 22, 42]. The structures that occur within these include DNA, RNA, and miRNA (Figure 1), but also purinergic ones, e.g., endonucleases or ectonucleases, which degrade ATP and hydrolyze AMP to adenosine, which causes binding and activation of adenosine A21 receptors and affects T cells [24, 37, 54, 55]. It has also been shown that microRNA found in EVs affects the development of exhausted T lymphocytes and the transfer of PD-1 receptors, making platelets an important element in patients with SLE, rheumatoid arthritis, and diabetes, among others [107]. Among the biologically active substances in platelet EVs affecting, among others, nucleic acids and TFs such as signal transducer and activator of transcription 3 (STAT3), signal transducer and activator of transcription 5 (STAT5), and PPARy, these compounds provide platelets with not only activating and regulatory effects but also transcriptional and metabolic effects, which builds and conditions good intravascular homeostasis, but also high potential of immune processes in the vessels [23, 68]. It is also assumed that platelet EV substances mainly increase the intravascular immune potential by increasing the activity of innate and acquired immunity, which is why they become essential elements in the pathogenesis and therapy of, among others, infectious, autoimmune, and neoplastic diseases [26, 42, 108]. These studies indicate that the biologically active substances of platelet EVs are also barriers—“barriers” for nucleases against nucleotide degradation and compounds affecting the stability of platelet microRNA. It is also indicated that platelet EVs, by affecting the programming of gene expression in PMN, MN, natural killer (NK) cells, APC, T and B lymphocytes, and stem cells, can be used in modifying immunity [83, 102, 109]. Biologically active compounds of these platelet organelles, taking part in the immunological synapse, determine their participation in local intercellular communication, as well as communication over long distances [29, 37, 108]. The substances of these organelles also affect the reproduction of male and female cells, as well as the course of pregnancy and activities related to the repair of bones, liver, and the central nervous system [23, 69]. They are also transcripts of specific immune mediators such as MMP family members—ADAM10, although they can also be carriers of infectious agents such as viruses and prions [29, 70, 102]. It has been shown that platelet EVs are also carriers of drugs or other therapeutic elements, mainly in cardiovascular and autoimmune diseases [6, 11, 42].

### 5.1. Platelet Exosomes in the Context of Intravascular Immunity

The structures known as exosomes are a subtype of EVs (Figure 1), also called MVs of the endosomal origin or small extracellular vesicles (sEVs). Their biogenesis involves production, release, and uptake by target cells [102]. They are formed as vesicles through budding from the endoplasmic reticulum and nuclear envelope into the inner membrane of the parent cell [102], and then, through fusion with the plasma membrane, form a multivesicular body, i.e., internal vesicles, which are released to the outside by exocytosis as exosomes [37, 102]. These structures are characterized by a spherical shape in the form of a cup with a diameter ranging from 20 to 100 nm or 30 to 200 nm and a density of 1.10–1.19 g/mL [23, 29, 102]. They were first discovered in 1983 in a sheep reticulocyte culture, and in 1987, they were given the name exosomes [38]. They contain biologically active substances (Figure 1), including compounds specific to their stem cell. These compounds play an important role in regulating the functions of the macroorganism, including the transfer of information between cells and the regulation of their functions, as well as in the pathogenesis of many diseases, including infectious diseases [31, 32, 35]. It has been shown that the substances of these platelet structures affect the homeostasis in blood vessels, including the blood clotting process, but also shape intravascular immunity by affecting the activity of T lymphocytes, Treg, TCD8, and NK lymphocytes, including their reprogramming, as well as intercellular communication [110, 111]. They play a role in immunity in infectious diseases and in hematological, autoimmune, and oncological disorders [95, 110]. It has also been registered that they assist in releasing proinflammatory mediators, which can lead to disturbances in the integrity of the vascular membrane and vessel “leakiness,” potentially contributing to the development of various pathological conditions [25, 112].

### 5.2. Platelet Ectosomes in the Context of Intravascular Immunity

Ectosomes, just like exosomes, are a subtype of EVs, which are also referred to as MVs (Figure 1). However, according to the Report of the International Society for Extracellular Vesicles from 2024 [99], it is indicated that the name ectosomes should be used conditionally. Ectosomes were first described as subcellular material derived from platelets [113]. It is currently assumed that they are vesicular fragments of the cytoplasm of these cells and, unlike exosomes or smaller vesicles, are referred to as internal vesicles [10, 23]. Ectosomes in the blood environment constitute the majority of platelet EVs [32, 114]. They arise directly from the cell membrane of these cells as a result of the process of “budding” or “shedding” of the cell membrane to the outside and are characterized by an irregular shape, density of 1.04–1.07 g/mL, and diameter of 100–1000 nm (Figure 1). The process of their release from platelets occurs as a result of the interaction of thrombin, protein complexes, complement components (C5b–C9) at an increased level of Ca2+ ions and during platelet activation occurring mainly via the integrin receptor αIIbβ3 (GPIIb/GPIIIa), ADP, and the receptor for the thrombospondin-related adhesive protein (TRAP) [23, 30, 32]. They also arise due to oxidative stress, inflammatory processes, apoptosis, and necrosis [32, 33]. P-selectin and differentiation molecules CD40 and CD63 are found on their surface, which can be used to distinguish them from other structures released from blood cells [3, 23, 35]. Platelet ectosomes (Figure 1) contain proteins, phospholipids (phosphatidylcholine, lysophosphatidylethanolamine, phosphatidylserine, phosphatidylinositol, sphingomyelin, and diacylphosphatidylethanolamine) and parental cell surface receptors, mRNA, microRNA, interleukins (IL1-β and IL-6), TFs (RUVBL2 protein–RuvB-like 2), signal transducers and activators of transcription (STAT3 and STAT5A), and free or embedded mitochondria, including mitochondrial DNA (mtDNA). These biologically active substances affect the activity of PMN, NK, natural killer T-cells (NKT), and B and T lymphocytes toward the secretion of cytokines, as well as vascular endothelial cells, which, together with other cellular elements of immunity in the blood, determines intravascular immunity and proper homeostasis and the development of inflammation [27, 32, 115]. The substances of these platelet structures also affect angiogenesis and neurogenesis and exhibit procoagulant activity, which may increase the risk of thrombus formation [32, 109]. An increase in the amount of these substances in platelet ectosomes in humans is recorded in the case of vascular endothelial damage, cardiovascular diseases, diabetes, obesity, antiphospholipid syndrome, sleep apnea, lupus erythematosus, rheumatoid and nonrheumatoid arthritis, as well as other immunological diseases [116, 117]. Their increased content has also been recorded in states of increased activity of the immune system, e.g. due to infections, the aging process of the body [23, 35], and in neoplastic processes [28, 34, 118]. Hence, it is indicated that the content of platelet membrane microfragments may also be a good indicator used in the diagnosis of various disease states [23, 69]. It should be added that a new subpopulation, defined as mitomicroparticles (MP), has recently been described within them [28]. These structures are characterized by free or embedded mitochondria, which stimulate oxidative phosphorylation in human cancer cells with lymphocytic leukemia, positively affecting the metabolism of these cells and supporting tumorigenesis. As indicated by the authors [42], these data may serve as a new therapeutic mechanism used in developing anticancer strategies.

## 6. Final Remarks and Summary

Although platelets are not cellular elements constituting the immune system, they exhibit extensive immunological activity. This feature of theirs, related to their immune role in blood vessels and their activities ensuring intravascular homeostasis, is mainly conditioned by their extremely rich set of biologically active substances in their granules and EVs. It means that these cells, as previously assumed, are not only cellular elements of blood that condition the maintenance of intravascular balance but also are very effective cellular components of blood that build and are responsible for the immune status in blood vessels, both in physiological and pathological conditions, including infections, autoimmune, and allergic diseases, as well as in cancer and mental diseases. Hence, due to this specific role of platelets in immunity, they are indicated today as elements also used in pharmacological treatment and therapy in the field of induction and development of inflammatory conditions, mainly resulting from the interaction of DAMPs and LAMPs [7, 46, 53]. This therapeutic direction of research on platelets, as it results from the cited literature, is currently associated with the development of substances (drugs) that mainly modulate their activity, including their action leading to dysregulation of the immune system, by influencing their aggregation or their killing, regulated by their specific receptors, such as the GPVI marker, P-selectin, and FcγRIIa, in cardiovascular diseases.

## Figures and Tables

**Table 1:** Biologically active substances found in platelet granules [7].

Granules and platelet elements	Substances found in platelet granulations
α granules	• Growth factors and mitogens: TGF-β, PDGF, EGF, IGF, VEGF, HGF, FGF, GM-CSF, PD-ECGF, endostatins
	• Coagulation system proteins: fibrinogen, vWF, integrins, factors V, VII, XI, XII, XIII, heparin-binding platelet factor h, protein S, kininogens, plasminogen, thrombin, alpha 2-antiplasmin
	• Cytokines: IL-1, IL-1α, IL-1β, IL-6, IFN-γ, TNFα, MIF, MMP 1, 2, 3, 9 i 14
	• Chemokines includes kinecidin: PF-4 (CXCL4), IL-8 (CXCL8), RANTES (CCL5), MCP3 (CCL-7), β-thromboglobulin, SDF-1 (CXCL12), MIP-1α (CCL3), TARC (CCL17), ENA-78 (CXCL5), MCP-1 (CCL2), NAP-2 (CXCL7), GRO-α (CXCL1), CXCR4, CCR4; CXCL16
	• Adhesion molecule: vitronectin, fibronectin, thrombospodin, ICAM-1, VCAM-1, selectin P (CD62P) and E and CD40 (CD40L), TLT-1
	• Inhibitors: TIMP, ADAM, α_2_-macroglobulin, α_2_-antitrypsin, α_2_-antiplasmin, antithrombin, PAI-1, inhibitor C1, COX-2, membrane glycoproteins, TFPI, protein S and C, nexins (amyloid protein beta A4), plasmin proteases, HMWK
	• Other molecules: defensins, PMP, β-lysine, IgG, IgE, IgM, albumin, transferrin, angiopoetin 1, 2, 3, APP, thymosin β-4, fibrinopeptide A i B, TRAP, calprotectin, HMBG-1, TXA2, PAF

δ granules osmophilic (dense granules)	• Amines and mediators: thrombin, serotonin, histamine, catecholamines (noradrenaline/adrenaline), epinephrine
	• Nucleotides: ATP, ADP, GDP
	• Other molecules: Ca^2+^ and Mg^2+^ ions, selectin P, glutamates, thrombocidins 1 and 2, polyphosphatases, pyrophosphates

Lysosomes (λ granules)	• Proteolytic enzymes: carboxypeptidases A and B, cathepsin A, D and E, colagenese, acid phosphatase, aryl sulphatase
	• Glycohydrolases: heparinase, β-glucoronidase, arylsulfatase, β-galactosidase, P-glycerophosphatase, β-glucosidase, D-glucosidase, β-fucosidase, α-mannosidase, D-mannosidase

Peroxisomes	• Catalase, peroxidase

T-granules	• TLR-9 receptors
	• Specific membrane proteins

*Note:* ADP, adenosine diphosphate; ATP, adenosine triphosphate; β-TG, beta thromboglobulin; C, complement; COX, cyclooxygenase; CCR4, C-C chemokine receptor type 4; CD40L (CD154), cluster of differentiation; CXCR4, C-X-C chemokine receptor type 4; ENA-78, epithelial neutrophil-activating protein 78; GDP, guanosine diphosphate; GRO-α, growth-regulated α; HMBG-1, high mobility group box 1; HMWK, high-molecular-weight kininogen; ICAM-1, intracellular adhesion molecule; IFN, interferon; Ig, immunoglobulin; IL, interleukin; MIF, macrophage migration inhibitory factor; MMP, matrix metalloproteinases; SDF-1, stromal cell-derived factor 1; TIMP, tissue inhibitors of metalloproteinase; TLT-1, (triggering receptor expressed on myeloid cells [TREM])-like transcript-1; TXA2, thromboxane A2.

Abbreviations: ADAM, a disintegrin and metalloproteinase; APP, amyloid precursor protein; EGF, epidermal growth factor; FGF, fibroblast growth factor; GM-CSF, granulocyte-macrophage colony-stimulating factor; HGF, hepatocyte growth factor; IGF, insulin-like growth factor; MCP-1, monocyte chemotactic protein 1; MIP-1α, macrophage inflammatory protein 1α; NAP-2, neutrophil-activating peptide-2; PAF, platelet-activating factor; PAI-1, plasminogen activator inhibitor 1; PD-ECGF, platelet-derived endothelial cell growth factor; PDGF, platelet-derived growth factor; PF-4, platelet factor 4; PMP, peroxisomal membrane protein; RANTES, regulated on activation, normal T expressed and secreted; TARC, thymus and activation-regulated chemokine; TFPI, tissue factor pathway inhibitor; TGF, transforming growth factor; TLR, toll-like receptor; TNF, tumor necrosis factor; TRAP, tryptophan regulated attenuation protein; VCAM-1, vascular cell adhesion molecule 1; VEGF, vascular endothelial growth factor; vWF, von Willebrand factor.

## Data Availability

Data sharing is not applicable to this article as no new data were created or analyzed in this study.
